# Early diagnosis of cardiac involvement in systemic lupus erythematosus via global longitudinal strain (GLS) by speckle tracking echocardiography

**DOI:** 10.15171/jcvtr.2018.40

**Published:** 2018-12-13

**Authors:** Farahnaz Nikdoust, Elham Bolouri, Seyed Abdolhussein Tabatabaei, Mahdi Goudarzvand, Seyedeh Tahereh Faezi

**Affiliations:** ^1^Department of Cardiology, Shariati Hospital, Tehran University of Medical Sciences, Tehran, Iran; ^2^Department of Physiology and Pharmacology, School of medicine, Alborz University of Medical Sciences, Karaj, Iran; ^3^Department of Rheumatology, Shariati Hospital, Tehran University of Medical Sciences, Tehran, Iran

**Keywords:** Systemic Lupus Erythematosus, Global Longitudinal Strain, Doppler Echocardiography, Left Ventricle

## Abstract

***Introduction:*** Systemic lupus erythematosus (SLE) myocarditis occurs in between 5% and 10% of patients with lupus. Global longitudinal strain (GLS) via speckle tracking echocardiography can detect cardiac involvement in patients suffering from SLE. We decided to determine the echocardiographic features and subsequent early diagnosis of cardiac involvement in patients with SLE utilizing the GLS index via speckle tracking echocardiography.

***Methods:*** In this cross-sectional study, we compared female patients with SLE of at least 2 years’ duration and healthy controls in terms of the left ventricular (LV) GLS via speckle tracking echocardiography. After data collection in both groups, the GLS index and the ejection fraction were evaluated.

***Results:*** We analyzed and compared the LV echocardiographic parameters of 33 patients with SLE (mean age=25.45±0.63 years) with those of 35 healthy controls (mean age=27±0.45 years). The apical 2-chamber view indicated a significant decrease in the LV GLS in the case group by comparison with the healthy controls (*P*=0.005). The LV GLS in the apical 3-chamber view was significantly lower in the case group than in the control group (*P*=0.006). The LV GLS in the apical 4-chamber view revealed no significant difference between the case and healthy control groups (*P*=0.2). While there was a significant difference between the case and control groups visà- vis the LV GLS (*P*=0.02), the LV ejection fraction measured with the Simpson method showed no significant difference between the 2 groups (*P*=0.96).

***Conclusion:*** GLS speckle tracking echocardiography is a noninvasive method with diagnostic and prognostic values; it may, therefore, be a sensitive marker for the diagnosis of myocarditis and other cardiac involvements in patients with SLE.

## Introduction

Systemic lupus erythematosus (SLE) is a chronic autoimmune inflammatory rheumatologic disease that can affect several organs such as skin, joints, and kidneys. One of the organs reported to be involved in SLE is the heart. Several cardiac diseases have been stated to be involved in patients with SLE; these diseases include pericarditis (the most common cardiac involvement), mitral regurgitation, tricuspid valve thickening and regurgitation, valvular vegetation (eg, Libman–Sacks endocarditis with a prevalence rate of 11%), myocardial dysfunction, and coronary arteries disease.^1-10^ Lupus myocarditis is a serious manifestation of SLE which occurs in 5% to 10% of patients with SLE.^11-16^

Cardiac involvement in SLE is frequently diagnosed with sensitive and noninvasive imaging methods such as echocardiography.^17-20^ Transthoracic echocardiography is applied to assess cardiac involvement in many autoimmune diseases such as SLE and rheumatoid arthritis. Most cardiac involvements in SLE are silent clinically, but they can cause significant illness and death.

Screening with transthoracic echocardiography can assess the systolic and diastolic functions of the heart and can measure the indices of global longitudinal strain (GLS) and ventricular wall-motion (velocity). In addition, speckle tracking echocardiography can be drawn upon to detect myocardial dysfunction and prevent early mortality.^21^ We, accordingly, sought to evaluate subclinical cardiac disease with the aid of speckle tracking echocardiography in patients suffering from SLE.

## Materials and Methods

This cross-sectional study recruited 33 female patients (age=20–30 years) with SLE of at least 2 years’ duration who referred to the Rheumatology Clinic of Shariati Hospital, Tehran University of Medical Sciences, Tehran, Iran. Patients with a history of Libman–Sacks endocarditis, a left ventricular ejection fraction (LVEF) of less than 55%, and heart failure were excluded. Thirty-five age- and sex-matched healthy controls were selected from the hospital staff. Following admission, a detailed history was obtained from the patients and clinical examinations were performed by experienced physicians. Demographic variables—including age, sex, height, and weight—were recorded. The patients and controls were also checked for the presence of classic cardiovascular risk factors such as hypertension, diabetes mellitus, dyslipidemia, smoking, and a family history of premature coronary artery disease. Additionally, patients with a history of valvular heart disease, arrhythmias, and known coronary artery disease were excluded. The clinical parameters checked encompassed the heart rate, systolic and diastolic blood pressures, a history of myocardial infarction or stroke, and congestive cardiac failure.

After the collection of the baseline demographic and clinical data, all the participants were evaluated with transthoracic 2D color echocardiography and speckle tracking echocardiography by a single echocardiologist using a Philips Dimensions ultrasound system.

The LV GLS was assessed in the apical 2-, 3-, and 4-chamber views. Echocardiographic parameters, including the diameter of the 4 chambers, were measured routinely. The LV systolic function and EF were measured using the Simpson method. The reference limits of all the echocardiographic parameters were defined according to the guidelines of the American Society of Echocardiography.^8,10^

### Statistical analysis

The normal distribution of the data in the 2 groups was assessed using the Kolmogorov–Smirnov test. The comparisons of the continuous variables between the 2 groups were analyzed with the SPSS software, version 18, using the Student *t* test or the Mann–Whitney U test. The results are expressed as means ± standard errors (SEs). A *P* value of less than 0.05 was considered the minimum significant difference of means.

## Results

**Figure 1:**
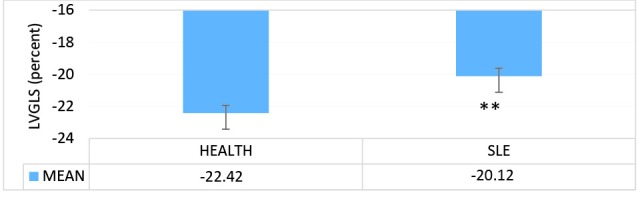
Apical 2-chamber view shows a significant decrease in the LV GLS in the patients with SLE (n=33) by comparison with the healthy controls (n=35) (*P*=0.005).

**Figure 2:**
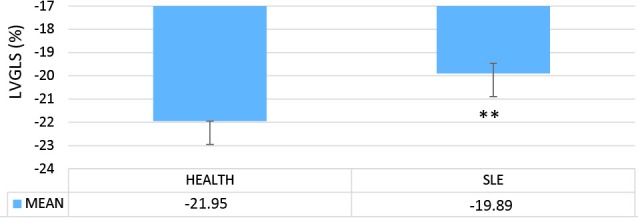
Apical 3-chamber view illustrates a significant decrease in the LV GLS in the patients with SLE (n=33) by comparison with the healthy controls (n=35) (*P*=0.006).

**Figure 3:**
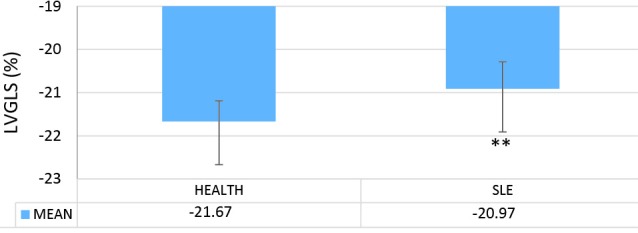
Apical 4-chamber view does not depict a significant difference in terms of a decreased LV GLS between the case group (n=33) and the healthy controls (n=35) (*P*=0.2).

**Figure 4:**
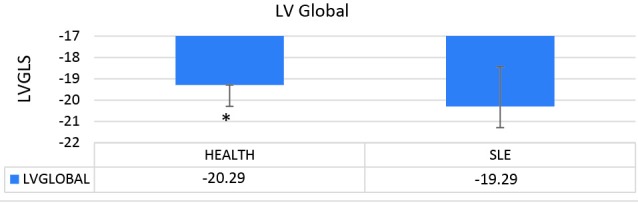
Measurement of the LV GLS shows a significant difference between the case group (n=33) and the control group (n=35) (*P*=0.02).

**Figure 5:**
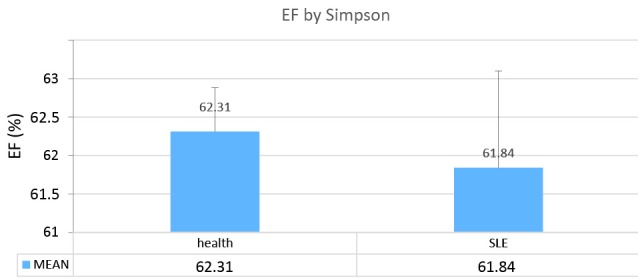
LVEF (%) measured with the Simpson method in echocardiography shows no significant difference between the patients with SLE (n=33) and the healthy controls (n=35) (*P*=0.96). The data are presented as mean ± SE.

LVEF, Left ventricular ejection fraction; SLE, Systemic lupus erythematosus.

In this study, we analyzed and compared the LV echocardiographic parameters of 33 patients with SLE (mean age=25.45±0.63 years) with those of 35 healthy controls (mean age=27±0.45 years). The results of the apical 2-chamber view showed a significant decrease in the LV GLS in the case group in comparison with the healthy controls (*P *= 0.005) (Figure 1). Similarly, in the comparison between the patients with SLE and the healthy controls in terms of the echocardiographic parameters, the LV GLS in the apical 3-chamber view was significantly lower in the case group than in the control group (*P *= 0.006) (Figure 2). In contrast, the LV GLS in the apical 4-chamber view revealed no significant difference between the case and control groups (*P *= 0.2) (Figure 3). Whereas the difference between the case and control groups as regards the LV GLS constituted a statistically significant difference (*P *= 0.02) (Figure 4), the 2 study groups were not statistically significantly different concerning the LVEF measured with the Simpson method (*P *= 0.96) (Figure 5).

The bars represent mean ± SE.

LV, Left ventricle; GLS, Global longitudinal strain; SLE, Systemic lupus erythematosus

**P<0.01 compared with the control group

The bars represent mean ± SE.

LV, Left ventricle; GLS, Global longitudinal strain; SLE, Systemic lupus erythematosus

**P<0.01 compared with the control group.

The bars represent mean ± SE.

LV, Left ventricle; GLS, Global longitudinal strain; SLE, Systemic lupus erythematosus

**P<0.01 compared with the control group.

The bars represent mean ± SE.

LV, Left ventricle; GLS, Global longitudinal strain; SLE, Systemic lupus erythematosus

* P<0.05 compared with the control group.

## Discussion

The availability of various treatment modalities has diminished the rate of death due to infections in patients with lupus. Nonetheless, death due to cardiac causes such as cardiomyopathy, arrhythmias, and heart failure plays a role as an independent factor in lupus.^4^ In an autopsy of patients with lupus, rates of cardiac involvement of between 40% and 50% have been reported, and between 7% and 10% of these cases of cardiac involvement are detected during the patients’ lifetime.^22,23^

One of the new methods for assessing the LV function with 2D speckle tracking echocardiography is GLS measurement. GLS measurement is a relatively new, accurate, and non-operator method to evaluate the function of the heart by comparison with the traditional methods of determining the EF.^24^

Studies have also shown that GLS measurement in comparison with the EF is a good predictor of mortality in patients with heart failure.^25,26^ In the onset of cardiovascular involvement in lupus, a significant change cannot be seen in the conventional EF measurement method. A measurement of the LV GLS may detect heart disease in lupus before the occurrence of a decreased EF.

In the present study, GLS and EF parameters in patients with SLE, but without cardiac symptoms, were compared with those of healthy subjects.

The results of the GLS assessment showed a significant decrease in the apical 2-, 3-, and global chamber views in the patients with SLE in comparison with the healthy subjects; nevertheless, no significant difference was found in the 4-chamber view results. Additionally, the LVEF finding in the patients with SLE indicated no significant decrease compared with the healthy controls.

In light of the above findings, it can be stated that an impairment in GLS can be used as an index for evaluating ventricular functions relative to the conventional EF measurement. In fact, GLS impairment has an early diagnostic and prognostic value.^16^ This finding is in line with the results of a study by Huang et al,^27^ who reported a reduction in the LV GLS in 2D echocardiography in their patients with lupus by comparison with their healthy subjects. A similar study, conducted in 2016 with the aid of speckle-tracking echocardiography to evaluate strain indices in patients suffering from lupus, indicated that the strain indices were decreased in the treatment-resistant lupus group compared with the non-resistant lupus group.^28^ These findings underscore the importance of strain in predicting the disease prognosis and response to treatment in patients with lupus.

A study in 2015 showed the predicting role of the GLS index in comparison with the EF index in chronic kidney disease. Cardiovascular disease is one of the causes of death in chronic kidney disease, which is mostly asymptomatic.^29,30^ Our research findings, in concordance with the findings of previous studies, indicate the predictive role of GLS in the LV function in patients with SLE. Several mechanisms have been suggested for cardiac involvement in patients with lupus. Indeed, chronic immune response, cytokine overactivity, and inflammatory progression contribute to cardiomyocyte apoptosis, ventricular dysfunction myocarditis, and finally heart failure. All these events increase the rate of cardiovascular mortality.^31-33^ Therefore, cardiovascular assessment in patients with lupus, even in non-symptomatic ones, appears to be crucial.

Given the limited research in this field in Iran, we conducted the current study in order to evaluate the early diagnosis of heart disease in patients with SLE by using the GLS index. We would, however, recommend further studies with the aid of GLS index.

### Limitations

The major limitation of the current study is the relatively small number of its participants. SLE is not a common disease and it is difficult, as such, to find patients with SLE. Another drawback of note is the single-center design of this study in a university hospital. We would recommend a multicenter study including a large number of patients with SLE in the different stages of the disease.

## Conclusion

Myocarditis and subsequent cardiac failure are a consequence of SLE. The GLS parameter in 2D speckle tracking echocardiography is a noninvasive cost-effective tool with a desirable diagnostic and prognostic value in patients with lupus myocarditis. GLS impairment was common in 2D speckle tracking echocardiography findings in our patients with lupus. Hence, it would be useful to consider screening 2D speckle tracking echocardiography in the routine care of patients with lupus to explore whether or not the GLS method would be cost-effective and beneficial for patients. Therefore, further investigations are needed on various aspects of cardiac involvement in lupus to determine the significance of GLS changes in autoimmune diseases such as lupus.

## Ethical approval

All the participants signed a written informed consent before enrolment in the study, and the study protocol was approved by the Board of Research and Ethics Committee of Tehran University of Medical Sciences.

## Competing interests

None.
